# Biogenic Synthesis of Silver Nanoparticles Using *Phyllanthus emblica* Fruit Extract and Its Inhibitory Action Against the Pathogen *Acidovorax oryzae* Strain RS-2 of Rice Bacterial Brown Stripe

**DOI:** 10.3389/fmicb.2019.00820

**Published:** 2019-04-26

**Authors:** Md. Mahidul Islam Masum, Mst. Mahfuja Siddiqa, Khattak Arif Ali, Yang Zhang, Yasmine Abdallah, Ezzeldin Ibrahim, Wen Qiu, Chenqi Yan, Bin Li

**Affiliations:** ^1^State Key Laboratory of Rice Biology and Key Lab of Molecular Biology of Crop Pathogens and Insects, Institute of Biotechnology, Zhejiang University, Hangzhou, China; ^2^Department of Plant Pathology, Bangabandhu Sheikh Mujibur Rahman Agricultural University, Gazipur, Bangladesh; ^3^Department of Botany, Jagannath University, Dhaka, Bangladesh; ^4^Institute of Biotechnology, Ningbo Academy of Agricultural Sciences, Ningbo, China

**Keywords:** *Phyllanthus emblica*, green synthesis, nanoparticles, characterization, antibacterial activity

## Abstract

Biogenic synthesis of silver nanoparticles (AgNPs) using plants has become a promising substitute to the conventional chemical synthesis method. In this study, we report low-cost, green synthesis of AgNPs using fresh fruit extract of *Phyllanthus emblica*. The biosynthesized AgNPs was confirmed and characterized by analysis of spectroscopy profile of the UV-visible and Energy dispersive spectrophotometer, Fourier transform infrared, X-ray diffraction pattern, and electron microscopy images examination. UV-visible spectra showed a surface resonance peak of 430 nm corresponding to the formation of AgNPs, and FTIR spectra confirmed the involvement of biological molecules in AgNPs synthesis. In spherical AgNPs, the particle size ranged from 19.8 to 92.8 nm and the average diameter was 39 nm. Synthesized nanoparticles at 20 μg/ml showed remarkable antimicrobial activity *in vitro* against the pathogen *Acidovorax oryzae* strain RS-2 of rice bacterial brown stripe, while 62.41% reduction in OD_600_ value was observed compared to the control. Moreover, the inhibitory efficiency of AgNPs increased with the increase of incubation time. Furthermore, AgNPs not only disturbed biofilm formation and swarming ability but also increased the secretion of effector Hcp in strain RS-2, resulting from damage to the cell membrane, which was substantiated by TEM images and live/dead cell staining result. Overall, this study suggested that AgNPs can be an attractive and eco-friendly candidate to control rice bacterial disease.

## Introduction

In recent decades, the development of new and effective antimicrobials against infections caused by antibiotic-resistant bacteria has been increasingly interested. Several studies have reported that nanoparticles made up of different noble metals like Ag, Cu, and Au, which can be applied to kill both resistant and nonresistant bacteria (Bindhu and Umadevi, 2014; Chidurala et al., 2016; Escárcega-González et al., 2018). In recent years, silver nanoparticles (AgNPs) have received a great deal of attention from many researchers working on multiple disciplines due to their unique features and a wide spectrum of applications (Sharma et al., 2009; Galdiero et al., 2011), for instance, in food science, medical, agriculture, and agricultural technologies (Kumar et al., 2008; Lü et al., 2009; Sastry et al., 2010; Oves et al., 2013; Aziz et al., 2015). Previous studies have demonstrated that AgNPs have potential antimicrobial activities against *Escherichia coli*, *Staphylococcus aureus*, and *Serratia marcescens* (Kim et al., 2007; Oves et al., 2013; Aziz et al., 2016). It also shows anti-nematodes (Mahmoud et al., 2016), anti-viral (Galdiero et al., 2011; Oves et al., 2013; Elbeshehy et al., 2015), anti-cancer (Oves et al., 2018; Aziz et al., 2019), and anti-inflammatory effects (Manikandan et al., 2017).

Several methods have been described for the synthesis of AgNPs, such as chemical reduction, microemulsions, radiation, hybrid methods, photochemical reduction and sonoelectrochemical, microwave-based systems and recently green synthesis route (Socol et al., 2002; Solanki and Murthy, 2010; Nadagouda et al., 2011; Iravani et al., 2014). However, these physiochemical methods, although some are durable and technically viable, are highly restricted in large-scale application due to the use of hazardous chemicals, high cost, high energy and time consuming, and difficulty in waste purification (Kowshik et al., 2003). Therefore, there is a growing need to use economical and environmentally safe and green synthesis routes that use non-toxic chemicals in the synthesis protocol of nanosilver. Alternatively, green synthesis route of AgNPs using several microorganisms, plants, and algae is natural, biocompatible, and environmentally safe methods (Bhattacharya and Gupta, 2005; Mohanpuria et al., 2008; Aziz et al., 2015).

Indeed, the use of plant materials can be more beneficial for nanosilver synthesis than bacterial and chemical methods because of no threat of bacterial and dangerous chemical contamination and less energy utilization with wider implications and easiness. Moreover, the green synthesis of AgNPs based on plant extract mechanisms alleviates the metal ions (Rai and Ingle, 2012) due to the presence of functional molecules such as phenol, terpenoids, ketones, carboxylic acids, aldehydes, enzymes, amides, and flavones (Prabhu and Poulose, 2012). Recent research reported that AgNPs have been synthesized using a variety of natural plants such as fruit extract of *Emblica officinalis* (Ankamwar et al., 2005), leaves extract of *Citrus limon* (Vankar and Shukla, 2012), green tea (*Camellia sinensis*) (Nakhjavani et al., 2017), *Coffea Arabica* (Dhand et al., 2016), neem (*Azadirachta indica*) (Ahmed et al., 2016), *Acalypha indica* (Krishnaraj et al., 2012), *Aloe vera* plant extract (Tippayawat et al., 2016), latex of *Jatropha gossypifolia* (Borase et al., 2014), root extract of *Morinda citrifolia* (Suman et al., 2013), *Phoenix dactylifera* (Oves et al., 2018), inflorescence extract of *Mangifera indica* (Qayyum et al., 2017), etc.

In this perception, *Phyllanthus emblica* L. (Family: Phyllanthaceae) commonly known as emblica/amla exhibits a striking assortment of shapes of development such as herbs and bushes, pachycaulous succulents, climbers, and drifting aquatics. The fruits of *Phyllanthus* spp. are widely used in preparation of traditional medicines in Southeast Asia due to unique properties such as rich antioxidant activity, anti-aging, antipuretic, and anti-inflammatory (Dang et al., 2011; Pientaweeratch et al., 2016; Manikandan et al., 2017). Moreover, this selection was provoked by the opportunity of inducing shape control of nanoparticles due to the potential sources of naturally occurring phytochemcals, (especially polyphenols, tannnis, ascorbic acides, flavonoids) in the fruit extract (Ramesh et al., 2015; Manikandan et al., 2017). Although many pharmacognosy and phytochemistry investigations have been successfully conducted in this plant, its opportunity as biocompatible materials for the production of AgNPs is still to be fully scanned. Therefore, we have used the *P. emblica* fruit extract to provide a biodegradable, natural product nanosilver without any risk of chemical toxicity, while the natural reserves will remain unaffected. In addition, there is no report on the inhibitory effect of AgNPs mediated by plants against rice pathogenic bacteria *Acidovorax oryzae* (Ao).

The Gram-negative bacteria Ao has wide host ranges within economically important monocotyledon plants such as cereal grain crops, and sugar crops (Hayward, 1960; Song et al., 2004; Masum et al., 2017). In particular, the individual strains of this pathogen can cause bacterial brown strip disease in rice, which has recently attracted considerable interest in China, for which contaminated seeds are the main sources for the spread of the disease to new plants over long distances (Shakya et al., 1985; Xie et al., 2011; Tian et al., 2015). Currently, bactericide is manily used to prevent and control bacterial diseases. However, the necessity for developing novel prevention and control strategies is increasing due to serious environmental pollution and bacterial resistance by the excessive use of chemicals in rice-growing countries across the world. Application of biosynthesized AgNPs is considerable interest in the field of agriculture because of their antioxidant and wide spectrum of antimicrobial activity along with their eco-friendly, biocompatible, and cost-effective nature. The nanoparticles are not only reported for improving plant improvement but exhibits different bactericidal mechanisms (Oves et al., 2013; Aziz et al., 2016; Qayyum et al., 2017; Mahawar and Prasanna, 2018). It is therefore of keen interest to scrutinize the inhibitory effect of synthesized AgNPs against Ao, which can be used in the field of nanotechnology as a cost-efficient, environmentally friendly and safe strategy.

Therefore, we aim to synthesize AgNPs using fresh fruit extract of *P. emblica* and evaluate their antibacterial efficacy against pathogen Ao strain RS-2 of rice bacterial brown stripe.

## Materials and Methods

### Materials

In order to obtain biosynthesized AgNPs, fresh fruits of *Phyllanthus emblica* were procured from the supermarket in Kunming City, Yunnan Province, China, and stored before use at 4°C. We acquired silver nitrate (AgNO_3_) from a company Sinopharm Chemical Reagent Co., Ltd. (Shanghai, China). Ao strain RS-2 previously isolated from natural diseased rice plants was collected from Plant Bacteriology Laboratory in Zhejiang University. The bacterial strain was routinely grown in Luria-Bertani (LB) medium consisting of tryptone 10 g, yeast extract 5 g, NaCl 10 g, ddH_2_O to 1000 ml and with/without agar 15 g, pH 7.0) at 30°C as described by Masum et al. (2017).

### Preparation of Extract From *P. emblica* Fresh Fruit

The fresh fruit extract of *P. emblica* fruit extract has been prepared following a procedure reported earlier (Ramesh et al., 2015; Manikandan et al., 2017) with slight modification. Briefly, the fresh fruits were carefully clean with sterilized double deionized water (ddH_2_O) and then chopped into small pieces and removed seeds. The sliced fruits were then finely macerated by a blender through sterile ddH_2_O to obtain 10% (w/v) fruit broth. The resulting extract was passed through a muslin cloth and then filter by Whatman No. 1 filters paper and kept at 4°C until use.

### Biosynthesis of AgNPs Using *P. emblica* Fruit Extract

**FIGURE 1:**
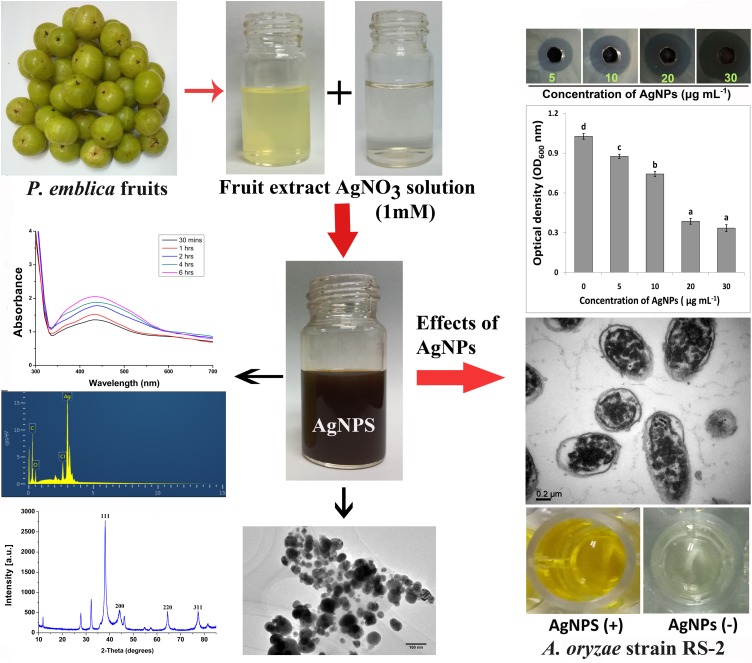
Schematic diagram for biosynthesis of AgNPs using fruit extract of *Phyllanthus emblica* and its inhibitory effect on Ao strain RS-2.

In the biosynthesis process of AgNPs, the effects of the quantity of fruit extract were assessed to intensify the synthesis route producing the metal nanoparticles. In a 100 ml aqueous solution AgNO_3_ (1 mM), various concentrations of aqueous fruit extract (2.5, 5, 10, and 15 ml) were added and boiled (65°C) for 20 min and then kept at room temperature under dark condition. To confirm that the synthesis of AgNPs was mediated by phytochemicals of *P. emblica* fruits, control flasks containing the mixture of aqueous solution of AgNO_3_ and sterile ddH_2_O were used. The reduction of silver ions was thus observed by changing the optical color in dark brown an taking into account the complete bio-reduction of Ag+, overnight samples of synthesized AgNPs were measured using UV-2550 Shimadzu Spectrophotometer (Shimadzu Corporation, Kyoto, Japan). The rapidly formed biosynthesized AgNPs were obtained by centrifugation at 10,000 rpm for 10 min in a centrifuge machine (JEOL, JEM-200EX; Tokyo, Japan) followed by carefully washed with sterile ddH_2_O and freeze-dried following the instruction of Alpha 1-2 LDplus (GmbH, Germany) and then stored at −80°C. Based on the fast reduction of AgNO_3_ into AgNPs, only the capable AgNPs sample prepared from 15 ml of fruit extract was used for further characterization using several methods including Fourier transform infrared spectroscopy (FTIR), X-ray diffraction (XRD), transmission electron microscopy (TEM), scanning electron microscopy (SEM), and energy dispersive X-ray (EDX) spectroscopy. All steps are shown sequentially in Figure 1.

### Characterization of the Synthesized AgNPs

Biosynthesized AgNPs due to the reduction of silver metal ions with aqueous *P. emblica* fruit extract was observed by UV-2550 Shimadzu Spectrophotometer (Shimadzu Corporation, Kyoto, Japan) operated with 1 nm resolution and wavelength of 200-800 nm. The dried sample’s FTIR spectrum was documented by the FTIR machine (Vector 22, Bruker, Germany) using ranges between 450 and 4000 cm^−1^ with a resolution of 4 cm^−1^. XRD pattern of the dried nanoparticles was obtained using Powder X-ray Diffractometer (Siemens D5000, Germany) as described in the instructions. In addition, X-MaxN Energy dispersive spectroscopy (EDS, Oxford Instruments, Oxford, United Kingdom) confirmed the presence of nanosilver elements at 20 keV. Scanning Electron microscopy (SEM) (SU8010, Hitachi, Japan) was used to characterize the shape of AgNPs. On the carbon-coated copper grid, a thin film of the dried samples was made by simply placing a little sample followed by drying for 5 min under the mercury lamp. A Field-Emission Scanning Electron Microscope (FE-SEM) images were used to study the size and morphology of AgNPs. Moreover, AgNPs have been structurally characterized in high resolution mode (HR-TEM) using FEI Technai F20 TEM instrument. To prepare a thin coat of the sample, a drop of silver solution was placed on the grid for 1 min, followed by solvent evaporation under vacuum and then placed sequentially in a grid box.

### *In vitro* Antibacterial Activity of AgNPs

Antimicrobial activity of synthesized AgNPs was evaluated against the pathogen Ao strain RS-2 of rice bacterial brown stripe by agar well diffusion technique as described by Elbeshehy et al. (2015) with little change. Briefly, 200 ml of bacterial suspension (approximately ∼1 × 10^8^ CFU/ml), previously overnight cultivated in LB broth at 30°C, was spread with 5 ml of LB agar medium on the top of solid LB agar medium in a Petri dish plate. Once the upper inoculated agar medium was air-dried, 40 μl of the final concentration of AgNPs from 5 to 30 μg/ml were loaded at the same distance on agar well (6 mm) and grown for 24 h at 30°C. The same amount of filter-sterilized *P. emblica* fruit extract was used as a control. Antibacterial activity was determined by averaging the diameter of inhibition zone formed around the center of each well. To determine the antibacterial activity, the diameter of the inhibition zone was measured around the whole. Two repeated experiment has been done following complete randomized design with three replications.

### Minimum Inhibitory Concentration (MIC) of AgNPs

The 5 ml AgNPs solutions have been adjusted by adding the stock of AgNPs to the half-strength LB broth in order to obtain a final concentration of AgNPs of 5, 10, 20, and 30 μg/ml, respectively. The MIC of AgNPs was determined by inoculating the 100 μl of bacteria cells of Ao strain RS-2 (∼approximately 1 × 10^8^ CFU/ml) into AgNPs solution of different concentrations, while the control was only sterile ddH_2_O (without AgNPs). The samples were then incubated at 30°C for 12 h at 180 rpm. The bacterial numbers in the samples were computed by reading the absorbance value at 600 nm using a ThermoMultiskan EX Microplate Photometer (Thermo Fisher Scientific, Waltham, MA, United States). With six replicates for each treatment, this experiment was repeated three times.

### Effect of Contact Time of AgNPs on Cell Survival of Ao Strain RS-2

The effect of incubation time of Ao strain RS-2 treated with AgNPs was evaluated by counting the viable cell spread on LB agar plates (Lou et al., 2011). In this procedure, AgNPs solutions were prepared by diluting the AgNPs stock (2 mg/ml) with sterile ddH_2_O to provide a final concentration of 20 μg/ml AgNPs, which has been listed as MIC against strain RS-2 by comparison with the other AgNPs concentrations tested. In order to achieve a final bacterial concentration of approximately 10^8^ CFU/ml, a newly prepared bacterial solution was supplemented with 5 ml of AgNPs solution; while in the control treatment, AgNPs was replaced with sterile ddH_2_O and then incubated for 3, 6, and 12 h in a shaker at 30°C. The bacterial suspensions were withdrawn from inoculated samples and diluted serially with sterilized ddH_2_O. A 100 μl of diluted samples were spread on LB agar plates and cultured at 30°C at 48 h. After incubation, the number of colony forming units (CFU) was enumerated to determine the cell survival activity while the average number of CFU was noted at the lowest dilution (Lou et al., 2011; Li et al., 2013). There were six replicates in each treatment and the experiment was done two times.

### Swarming Motility Assay

The effect of AgNPs on Ao strain RS-2 swarming motility was assayed on LB plates supplemented by 0.7 percent (w/v) agar as previously described by Dong et al., 2016). In the center of each swarming plate containing AgNPs (20 μg/ml), 5 μl of strain RS-2 suspension (approximately 1 × 10^8^ CFU/ml) were dropped, while the plate without AgNPs was served as control and then incubated for 3 days at 30°C. To evaluate the swarming motility, the colony diameter of strain RS-2 was assessed as reported in our previous study (Masum et al., 2017). The motility assay was repeated three times with six replications.

### Biofilm Inhibition Assay

The inhibitions of bacterial biofilm formation by AgNPs were performed using the 96-well microtitre plate method as described Masum et al. (2018) with slight modification. Briefly, the overnight cell suspension of Ao strain RS-2 was re-cultured into a fresh LB broth at shaker to obtain the mid-exponential growth. Thereafter, 100 μl of bacterial cells (approximately ∼1 × 10^8^ CFU/ml) was inoculated onto each well with AgNPs concentration of 20 μg/ml, while sterile ddH_2_O were used as a control. The plates were kept without agitation at 30°C for 24 h of adhesion. Culture media from each well were then removed and washed gently with sterile ddH_2_O. At room temperature, 100 μl solution of crystal violet (0.1%, w/v) was added to stain the biofilm in the well of plate and incubate for 45 min. The unattached violet crystal solution was discarded three times from the plate with sterile ddH_2_O. Bacterial biofilm was determined by measuring the absorbance at 570 nm after dissolving the CV stain with 125 μl of acetic acid (33%, v/v) using a SPECTRAmax^®^PLUS384 Microplate Spectrophotometer. With 12 replications for each treatment, this experiment was repeated three times independently.

### Live/Dead Cell Staining

To observe damage and intact membranes in bacterial cells exposed to 20 μg/ml AgNPs, live/dead staining technique was used following the protocol of BacLight bacterial viability kit (Invitrogen). There are two nucleic acid stains in the kit namely (i) a red-fluorescent (propidium iodide stain), and (ii) a green-fluorescent (SYTO 9 stain). To verify the validity of the kit, live bacteria as a negative control, and the dead bacteria samples treated by isopropanol were stained, respectively, according to the description of the kit’s protocol. Fluorescence in the sample was then detected using the Olympus inverted confocal microscope as described previously (Cui et al., 2014).

### Transmission Electron Microscopy (TEM) Analysis

For TEM analysis, Ao strainRS-2 has been prepared as mentioned in the previous studies (Li et al., 2013; Dong et al., 2016). Briefly, 1000 μl suspension of Ao strainRS-2 (approximately 1 × 10^8^ CFU/ml) was inoculated with AgNPs, to a final 20 μg/ml of AgNPs. The treated sample was incubated at rotary 160 rpm shaking at 30°C for 8 h. The bacterial suspension without AgNPs served as control. The bacterial cells were washed twice after centrifugation with a solution containing 0.1 mol/l phosphate-buffered saline (PBS) (pH 7.2) and fixed in glutaraldehyde [2.5% (v/v) in 0.1 M PBS]. The samples were then post-fixed with osmium tetroxide [1% (w/v) in 0.1 M PBS] for 1 h at room temperature and rinsed with the 0.1 M PBS buffer three times, followed by dehydration with a series of ethanol solutions including 50, 70, 80, 90, 95, and 100%. A low-viscosity medium of Spurr resin was used to embed the specimen which has been incised with a diamond knife on Supernova Ultramicrotome (Astria). The section stained with uranyl acetate and alkaline lead citrate was monitored in TEM of Model JEM-1230 (Japan) following the standard procedures.

### Secretion of Effectors Protein Hcp (Hemolysin Coregulated Protein)

The secreted Hcp protein of Ao strain RS-2were measured by Enzyme-linked immune sorbent assay (ELISA) experiment as described in our previous studies (Li et al., 2014; Masum et al., 2017). The ELISA was conducted in a standard 96 microtiter plate (Corning-Costar Corp., Corning, NY, United States) as described by Slutzki et al. (2012). Briefly, 150 μl of filtered antigen was pipetted to coat the microtiter plates and incubated overnight at 4°C followed by washing with wash buffer. After blocking the plates with a blocking buffer of 175 μl/well, it was kept at 37°C for 1 h and decanted. In order to detect Hcp protein of Ao strainRS-2 treated with or without AgNPs at 30 μg/ml, the Hrp-conjugated Goat Anti-Rabbit IgG polyclonal antibody was used at a dilution of 1:5000. The value of optical density (OD_450_) was registered using a microplate reader (Multiscan microtiter plate reader) set at 450 nm.

### Statistical Analysis

The experimental data were analyzed using the SPSS software package SPSS 21 (United States) and the mean values of the treatments were grouped by selecting the LSD (least significant difference) tools. Data are represented as the average values with standard error of at least three values of each independent experiment.

## Results

### Synthesis and Characterization of the Synthesized Nanoparticles

**FIGURE 2:**
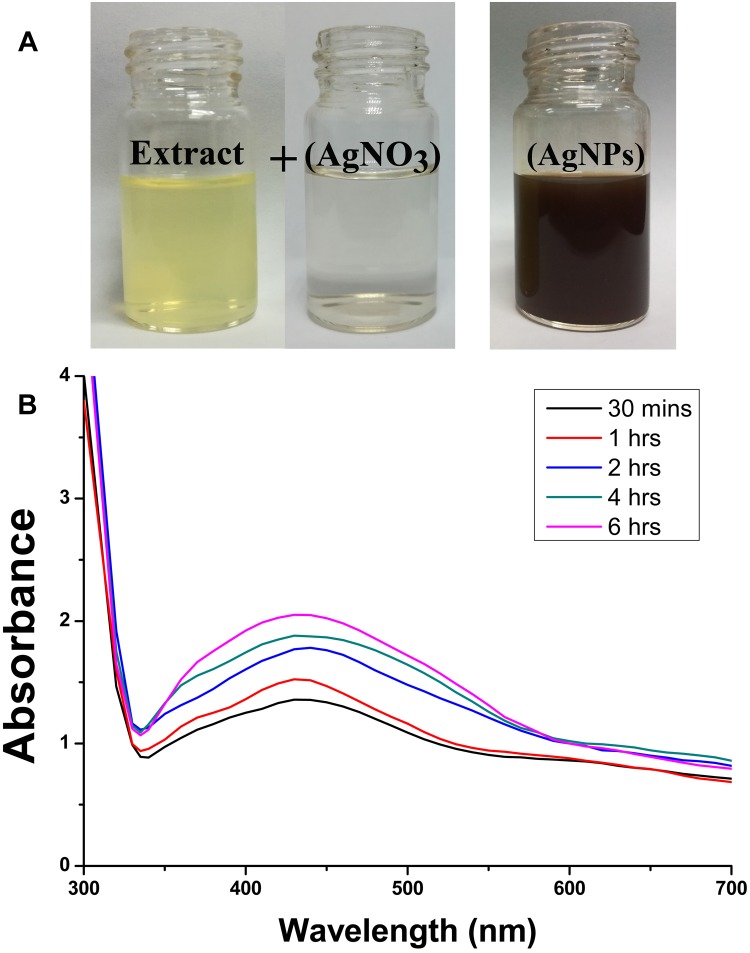
Confirmation of biosynthesized silver nanoparticles (AgNPs). **(A)** Color changes in the *Phyllanthus emblica* fruit extract (PFE) after addition of silver nitrate (1 mM aqueous solution of AgNO_3_) for nanoparticles synthesis; **(B)** UV–visible spectrum of AgNPs synthesized by phytochemical agents in the PFE. The peak at 430 nm corresponds to the surface plasmon resonance of AgNPs.

In order to standardize the nanoparticles synthesis route, different quantities of *P. emblica* fruit extract varied from 2.5, 5, 10, 15 ml with 100 ml aqueous solution of AgNO_3_ (1 mM) were tested in this study. After 30 min, the dissolution of the 15 ml fruit extract caused the rapid change in color from light yellowish to dark brown, indicating the fast reduction of Ag^+^ to Ag^0^ in AgNO_3_ solution (Figure 2A), while the color in other samples was changed after incubation for 2–8 h in a dark room and the control sample remained colorless. Furthermore, the synthesis of AgNPs in the solution was confirmed by the results of UV-visible spectrophotometers, which exhibited a spectrum of surface plasmon resonance (SRP) ranging from 430 to 436 nm of absorption band (Figure 2B). However, it could be noted that AgNPs was also synthesized with 2.5 ml of fruit extract in 100 ml AgNO_3_ solution. Indeed, the SPR spectra of AgNPs derived from the higher concentration of fruit extract showed a sharper and strong absorption band at 430 nm (Figure 2B). Moreover, the UV-vis spectra results showed an increase in the absorbance intensity of the reaction mixture with time and the solution was stable after 24 h of incubation, which indicates the completion of nanoparticles formation in solution. Therefore, the AgNPs mediated by the mixture of 15 ml fruit extract and 100 ml AgNO_3_ solution was freeze-dried and used for further studies.

**FIGURE 3:**
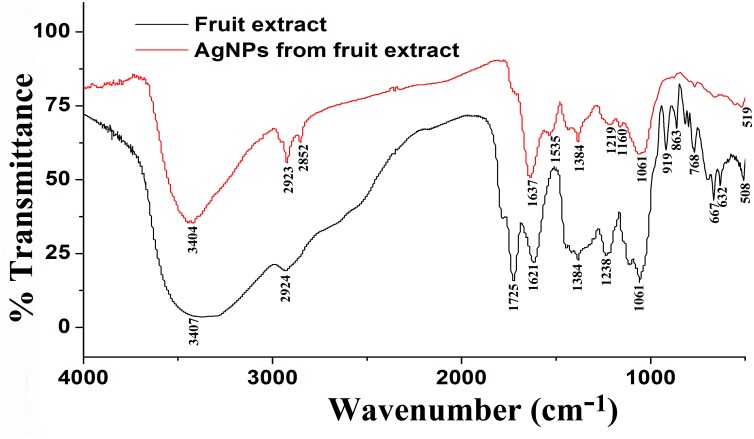
Fourier-transform infrared spectrum of biosynthesized AgNPs (up) and fruit extract of *Phyllanthus emblica* (down) after 24 h of incubation.

In addition, Figure 3 shows the FTIR spectra of biogenic AgNPs derived from *P. emblica* fruit extract after reaction with AgNO_3_ and fruit extract control without AgNO_3_. The FTIR data indicates the marginal shift in the peak position of spectra as depicted in Figure 3. The spectral analysis reveals the number of functional biological group responsible for stabilization of nanoparticles, which acts as capping or stabilizing agents. FTIR measurement based on AgNPs mediated by fruit extract revealed different absorption peaks at 3404, 2923, 2852, 1637, 1535, 1384, 1219, 1160, 1061, and 519 cm^−1^. In case of AgNPs, a very strong absorption peak shifted toward a lower wave number was observed at 3404 cm^−1^, which indicates the binding of silver ion (Ag^+^) with hydroxyl and or amine groups in the *P. emblica* fruit extract. Other bands figured at about 2923 and 2852 cm^−1^, are also remarkable because of the stretching vibration of hydrocarbon (C–H) bonded of alkenes, while the peak at 1637 cm^−1^ is also predominant and represents the involvement of amide-I bond (–C = O) of proteins as a capping agent and stabilization of AgNPs. Moreover, the band at 1621 cm^−1^ in fruit extraction was due to the presence of amide I vibrations, which was shifted to 1535 cm^−1^ in AgNPs due to the proteins that may have been linked to AgNPs by the amine groups. The peak observed around 1384 cm^−1^ in AgNPs spectra extract that could be assigned to C–H symmetric vibrations and same peak was also observed in fruit extract. The spectral peak at 1238 cm^−1^ in extract (shifted to 1219 cm^−1^ in AgNPs) was found by the C–C stretching vibration (Figure 3).

**FIGURE 4:**
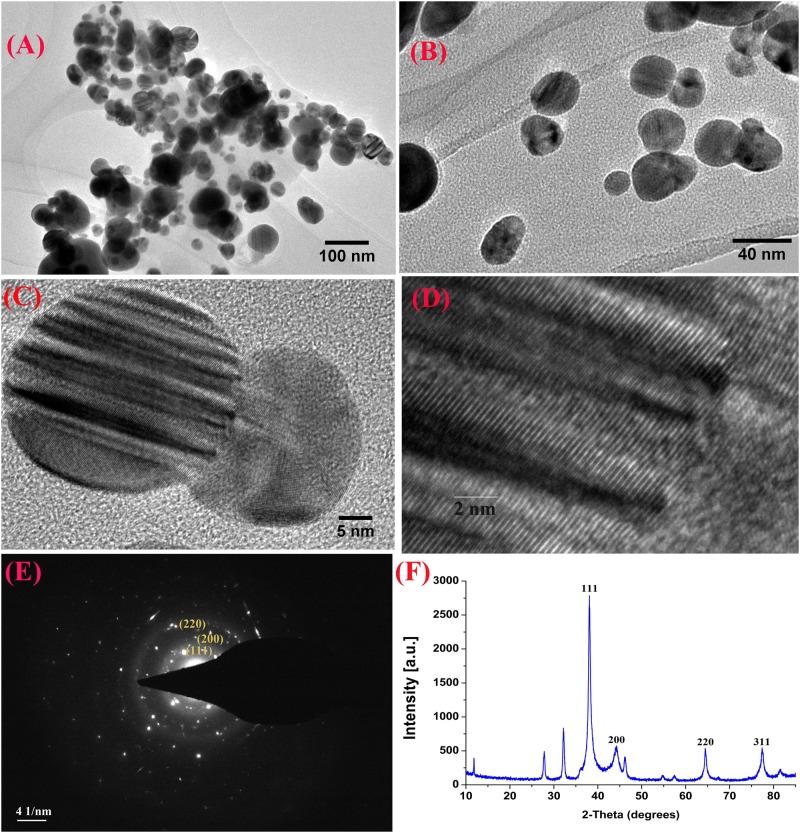
TEM images of the biosynthesized silver nanoparticles (AgNPs) **(A,B)**; HRTEM image with lattice fringe **(C,D)**; corresponding SAED pattern **(E)**; and X-ray diffraction patterns of synthesized AgNPs **(F)**.

**FIGURE 5:**
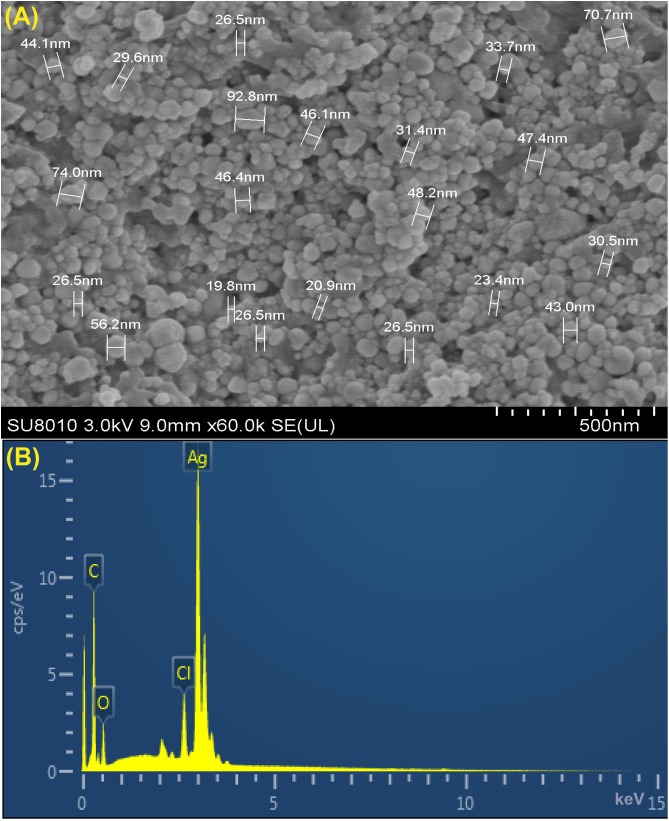
**(A)** SEM image of silver nanoparticles (AgNPs) synthesized by reducing 1 mM AgNO_3_ using fresh fruit extract of *Phyllanthus emblica*. A representative SEM image recorded from a thin film of synthesized AgNPs prepared on carbon-coated copper grid and estimation of nanoparticles diameter. **(B)** Energy Dispersive Spectroscopy (EDS) patterns of synthesized AgNPs; EDS spectra recorded from a film of synthesized AgNPs with different X-ray emission peaks labeled.

Furthermore, XRD analysis confirmed the crystalline nature of mediated AgNPs based on the emission peaks of 2θ = 38.178, 44.428, 64.583, and 77.639°, corresponding to the silver crystal planes (111), (200), (220), and (311), respectively, (Figure 4F). The TEM images clearly showed that most AgNPs were highly mono-dispersed in spherical shapes (Figure 4A,B),which was in conformity with the SEM image (Figure 5A). Moreover, the HR-TEM images (Figure 4C,D) of a biosynthesized silver nanoparticle revealed crystalline nature of the particles showing the lattice fringe quite clearly. The bright circular spots in SAED pattern (Figure 4E) demonstrated the (111), (200), and (220) planes, which also revealed the crystalline nature of the particles formed. These data are in lines with the XRD results obtained. The size distributions of the AgNPs varied between 19.8 and 92.8 nm with a mean value of 39.1 nm (Figure 5A). EDX instrument furthermore confirmed the existence of the silver element in the synthesized AgNPs (Figure 5B).

### *In vitro* Antimicrobial Activity of AgNPs

**FIGURE 6:**
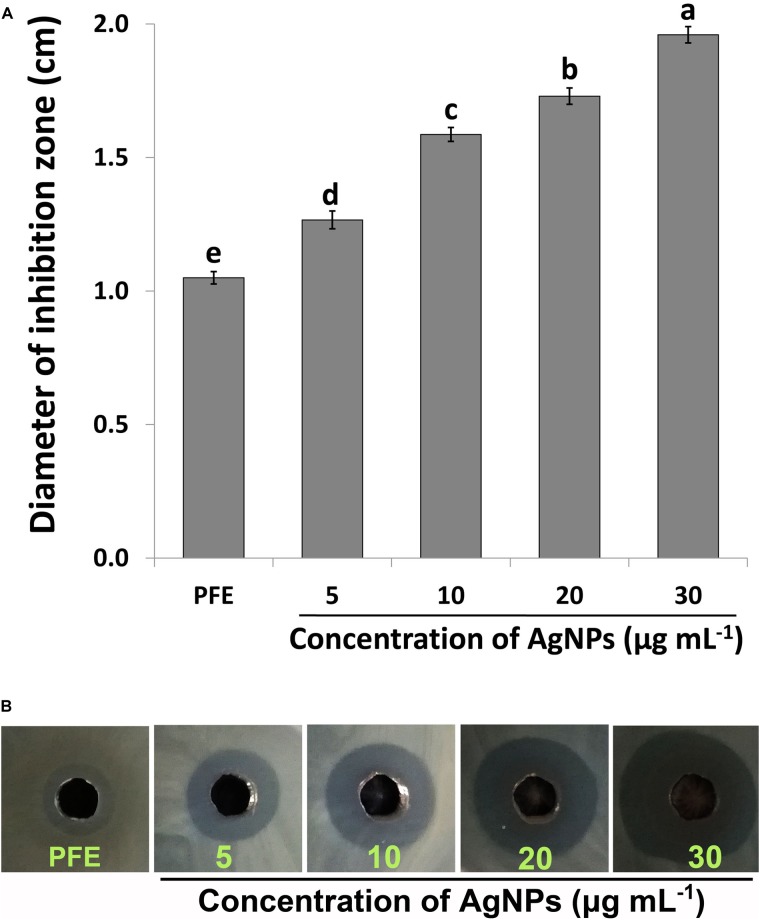
*In vitro* inhibitory effect of silver nanoparticles (AgNPs) synthesized by *Phyllanthus emblica* fruit extract (PFE) against *Acidovorax oryzae* strain RS-2. **(A)** Diameter of bacterial growth inhibition (cm) caused by PFE and different concentration of AgNPs; Vertical bars represent standard errors of the means (*n* = 3). Bars followed by the same letter(s) are not significantly different (*P* ≤ 0.05). **(B)** Bacterial growth inhibition zone achieved in agar well diffusion method by PFE and concentration of AgNPs at 5, 10, 20, and 30 μg/ml.

The biosynthesized AgNPs exhibited good sensitivity response at the four different concentrations against Ao strain RS-2 compared to *P. emblica* fruit extract (PFE) after 24 h of incubation in the agar media (Figure 6). The inhibition zone diameter increased by increasing the concentration of AgNPs against strain RS-2, which varied from 1.27 to 1.96 cm (Figure 6B). The largest inhibition zone was achieved by a concentration of 30 μg/ml, followed by 20 μg/mlof AgNPs (Figure 6A). In contrast, the control PFE showed the smallest inhibition zone (1.05 cm) compared to the addition of AgNPs treatments (Figure 6A). Although antibacterial activity was detected in the control containing only AgNO_3_ (data not shown), it was significantly increased by the addition of AgNPs. Taken together, these results suggested that the synthesized AgNPs showed an excellent antimicrobial activity against strain RS-2.

### Minimum Inhibitory Concentration (MIC) of AgNPs Against Ao Strain RS-2

**FIGURE 7:**
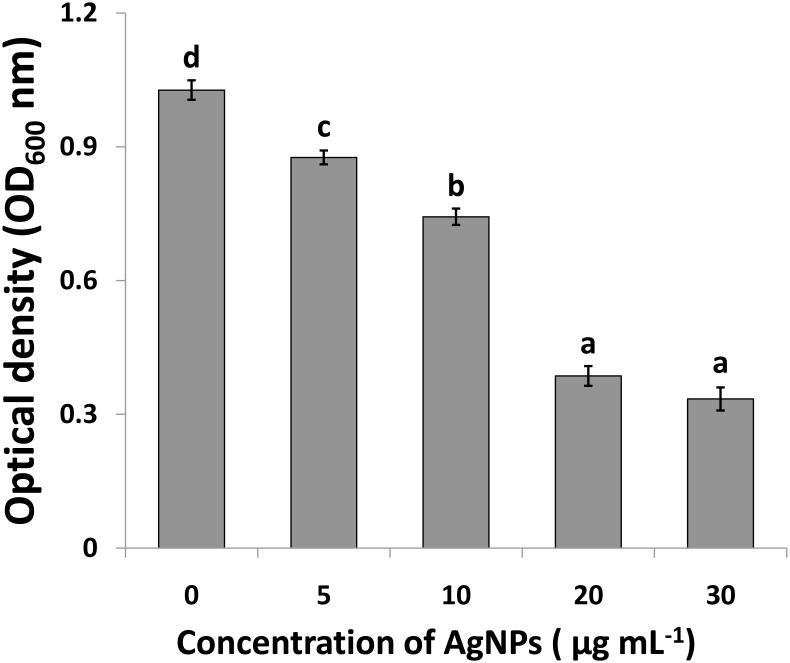
Determination of minimum inhibition concentration of silver nanoparticles (AgNPs) mediated by *Phyllanthus emblica* fruit extract against *Acidovorax oryzae* strain RS-2. Vertical bars represent standard errors of the means (*n* = 6). Bars followed by the same letter(s) are not significantly different (*P* ≤ 0.05).

Compared to the control, the results of this study showed that AgNPs had a noticeable antimicrobial activity against Ao strainRS-2 after 12 h of incubation (Figure 7).The antimicrobial activity was varied at different levels of AgNPs. In general, the biosynthesized AgNPs concentrations of 5, 10, 20, and 30 μg/ml caused 14.70, 27.65, 62.41, and 67.43% reduction in the OD_600_ values, respectively, while a maximum value of OD_600_ (1.027) was observed in strain RS-2 in the absence of AgNPs (Figure 7). However, there was no significant difference between the concentration of 20 and 30 μg/ml in the antibacterial activities of AgNPs, which indicated that Ao strain RS-2 is highly susceptible to both AgNPs concentrations.

### Effect of Contact Time of AgNPs on Cell Survival of Ao Strain RS-2

To investigate whether the incubation time affects the antimicrobial activity of AgNPs; an experiment was conducted by enumerating the surviving cell of Ao strain RS-2 at different incubation period up to 12 h. Results showed that the initial bacterial survival of strain RS-2 in sterile ddH_2_O was 8.19 log CFU/ml as a control, while the bacterial survival remained stable with the increase in the contact time (Table 1). However, in the AgNPs treatments (20 μg/ml), the cell survival of strain RS-2 decreased by 1.40, 3.65, 4.09, and 4.97 log CFU/ml as compared with the corresponding control after 1.5, 3.0, 6.0, or 12.0 h of contact time, respectively. Obviously, in the presence of AgNPs, cell survival was decreased with the increase in contact time (Table 1).

**Table 1:** Effect of contact time of AgNPs on the antibacterial activity against *Acidovorax oryzae* strain RS-2.

Contact time (h)	Bacterial survival (log_10_ CFU/ml)	
	Control	AgNPs^a^
0.0	8.19 ± 0.010 a^b^	8.19 ± 0.010 e
1.5	8.18 ± 0.013 a	6.78 ± 0.089 d
3.0	8.16 ± 0.012 a	4.51 ± 0.056 c
6.0	8.12 ± 0.022 a	4.03 ± 0.024 b
12.0	8.09 ± 0.027 a	3.12 ± 0.041 a

*^a^Concentration of AgNPs solution was 20 μg/ml. ^b^Data from the two repeated experiments with six replications were pooled because there was no significant difference between repeat experiments. Means in a column followed by the same letter are not significantly different according to the LSD test (P ≤ 0.05).*

### Swarming Motility

**FIGURE 8:**
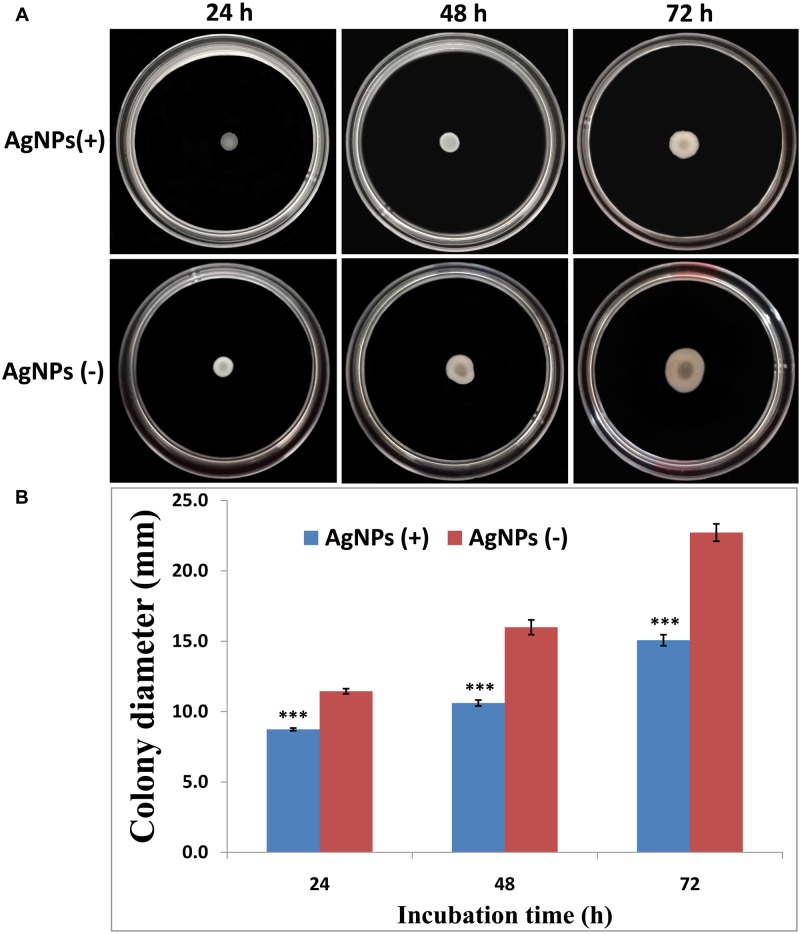
Effect of silver nanoparticles (AgNPs) mediated *Phyllanthus emblica* fruit extract on the swarming motility of *Acidovorax oryzae* strain RS-2. The concentration of AgNPs is 20 μg/ml. ^∗∗∗^*P* < 0.001. Error bars represent the standard error of the mean (*n* = 6). **(A)** Bacterial swarming motility was determined by measuring the diameters of bacterial colony on the plates from three independent experiments; **(B)** Colony diameter at different incubation time.

The effects of AgNPs on the bacterial movement were examined by evaluating the diameter of vicinity covered by Ao strain RS-2 on LB agar plates supplied with AgNPs (20 μg/ml). Results of experiment showed that colonies measurement of strain RS-2 were 11.4, 16.0, and 22.7 mm in the absence of AgNPs after incubation of 24, 48, and 72 h (Figure 8A). Interestingly, the swarming ability of strain RS-2 was significantly inhibited by the incubation with AgNPs at different time (Figure 8B). As a result, the colony diameter of strain RS-2 were 8.7, 10.6, and 15.1 mm after 24, 48, and 72 h of incubation with AgNPs, which were decreased by 23.68, 33.66, and 33.67%, respectively, compared with the corresponding control (Figure 8). These results showed that the AgNPs had a significant effect on the swarming motility of Ao strain RS-2 at a concentration of 20 μg/ml regardless of the incubation period.

### Biofilm Formation

**FIGURE 9:**
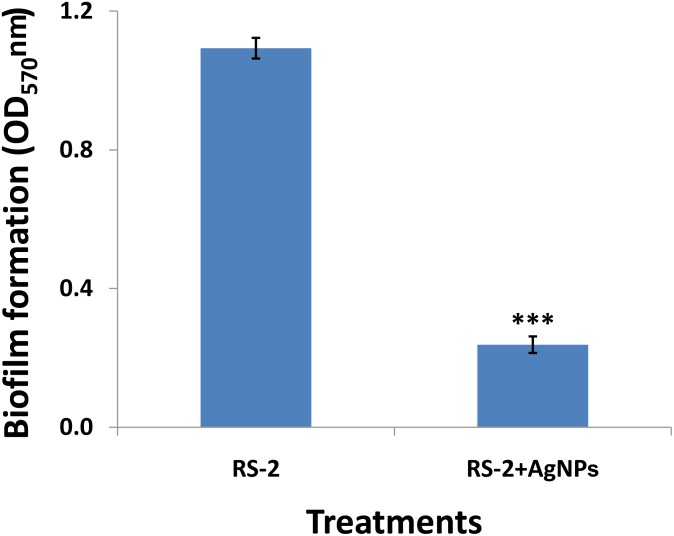
Effect of silver nanoparticles (AgNPs) mediated *Phyllanthus emblica* fruit extract on biofilm formation of *Acidovorax oryzae* strain RS-2 after 24 h incubation at 30°C for 24 h without agitation. Vertical bars represent standard errors of the means (*n* = 6). The concentration of AgNPs is 20 μg/ml. ^∗∗∗^*P* < 0.001.

After 1 day’s incubation at 30°C without stirring, the biofilm formation of strain RS-2 was quantified and displayed a significant inhibition effect when we exposed a concentration of 20 μg/ml of AgNPs in microtitre plates, compared with control (Figure 9). Indeed, the OD_570_ value of Ao strain RS-2 was 0.129 without AgNPs, while the strain RS-2 treated with AgNPs had a lower OD_570_ value (0.043). In general, the biosynthesized AgNPs caused 66.64% reduction in the OD_570_ value of Ao strains RS-2 as compared to the control (Figure 9).

### Live/Dead Cell Staining

**FIGURE 10:**
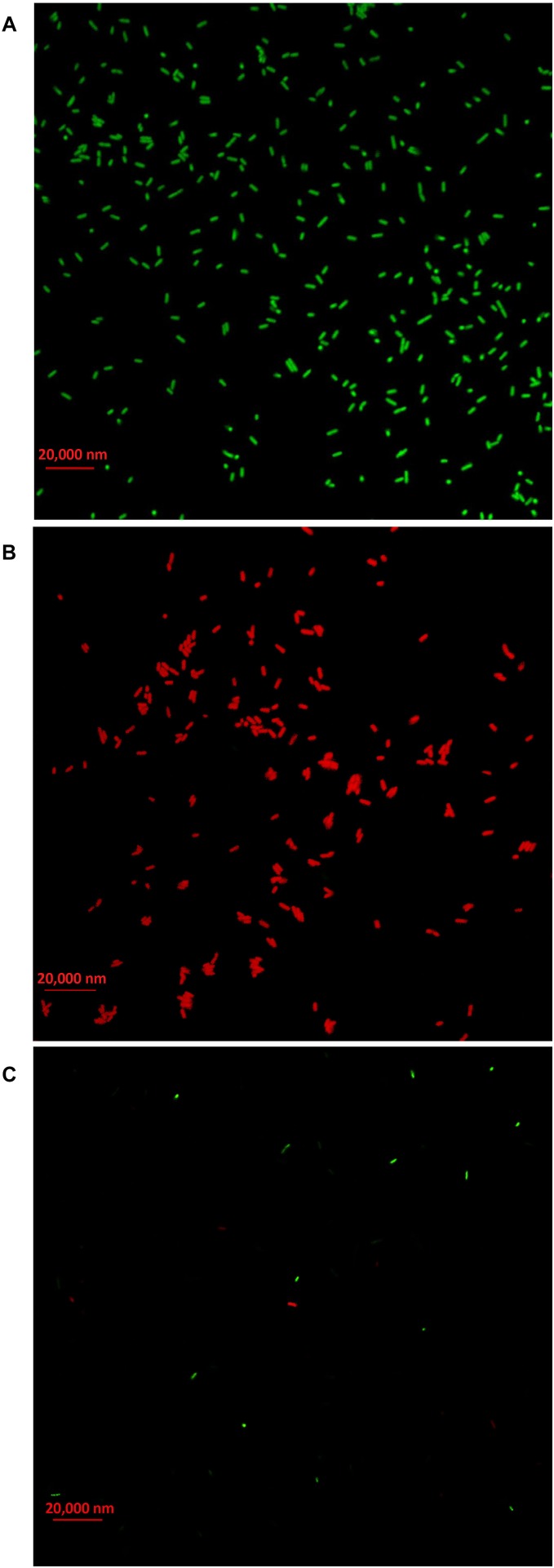
Live/dead cell staining analysis of *Acidovorax oryzae* strain RS-2 cells exposed to 20 μg/ml of silver nanoparticles (AgNPs) for 6 h. Staining were carried out using live/dead BacLight bacterial viability kit (Invitrogen, Carlsbad, CA, United States), and visualized by fluorescence microscopy. Green fluorescence is representative of live bacteria with intact membranes, while red fluorescence is representative of dead bacteria. **(A)** Live bacteria in negative control (without AgNPs); **(B)** Dead bacteria in negative control (Heat); **(C)** Bacteria in AgNPs treatment.

In order to clarify the antimicrobial mechanism of AgNPs, the live/dead bacterial cells of Ao strain RS-2 were stained after 8 h of treatment. Without AgNPs, staining results in live bacteria clearly showed intact membranes, which can be induced from fluoresce green (Figure 10A), while fluoresce red dead cells were detected after heated the bacteria (Figure 10B). Interestingly, after exposing the strain RS-2 to AgNPs (20 μg/ml), some bacterial cells were monitored to fluoresce green (Figure 10C). But, when AgNPs was treated, the number of green fluorescent cells of strain RS-2 decreased significantly, suggesting an inhibition of bacterial growth and replication. Taken together, these results indicate that AgNPs had a bactericidal effect on strain RS-2.

### Damage of Bacterial Cells by the Synthesized AgNPs

**FIGURE 11:**
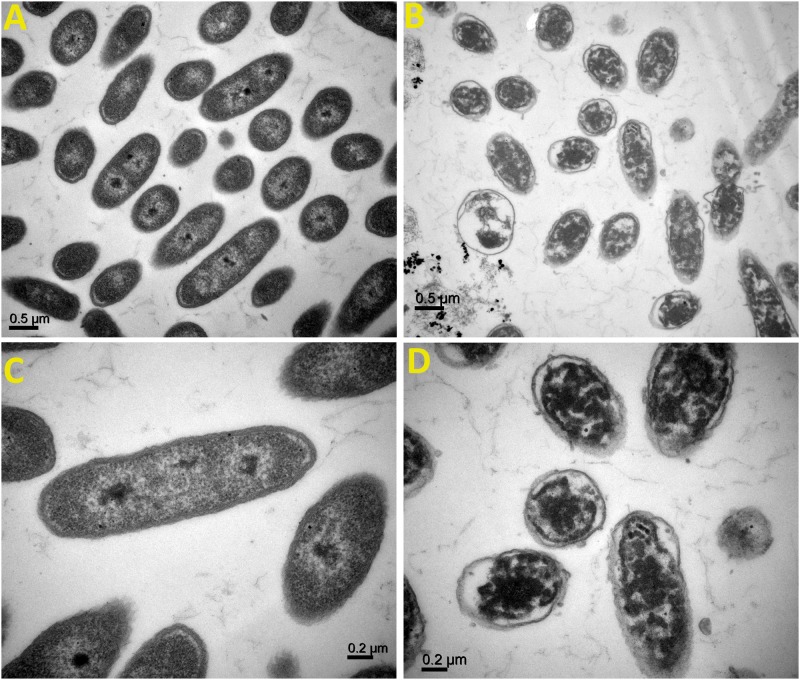
Transmission electron microscopic observation of *Acidovorax oryzae* strain RS-2 treated without **(A,C)** and with **(B,D)** biosynthesized silver nanoparticles (AgNPs)at 20 μg/ml. Scale bar in **(A,B)** = 0.5 μm; in **(C,D)** = 0.2 μm.

TEM analysis of Ao strain RS-2 was used to study the effect of AgNPs on the major structural damage of bacterial cells. TEM results of strain RS-2 indicated that the membranes of untreated cells were intact with uniformly distributed cytochylema and electron-dense material throughout the bacterial cytoplasm (Figure 11A,C). After treatment with AgNPs, however, the cell wall and cytoplasmic membrane of strain RS-2 became wrinkle and abnormal (Figure 11B,D). Besides, the synthesized AgNPs badly ruptured the part of the cell wall, therefore, leading to leaching out of nutrient and nucleic material swollen cell structure and caused the death of the bacterium, and there was evidence of dead cells in live/dead staining images.

### Secretion of Effector Protein Hcp

**FIGURE 12:**
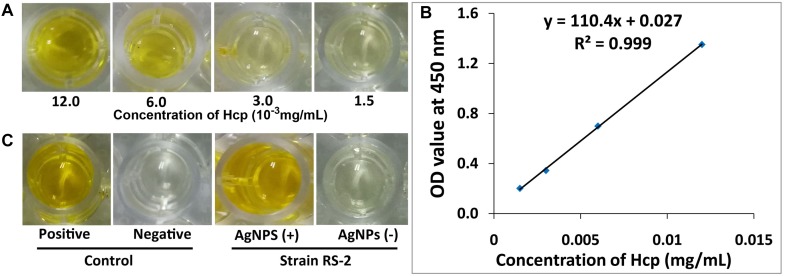
Effect of silver nanoparticles (AgNPs) mediated *Phyllanthus emblica* fruit extract on the secretion of Hcp protein in *Acidovorax oryzae* strain RS-2. **(A)** ELISA of Hcp protein at different concentrations; **(B)** Standard curve, the negative reaction (P/N value < 1.5); **(C)** ELISA measurement of Hcp secreted by strain RS-2. The positive reaction (P/N value ≥ 1.5); The purified Hcp-His fusion protein and His protein were used as the positive and the negative control, respectively. The experiment was conducted three times with three replicates.

Effects of AgNPs on effector protein Hcp secretion in Ao strain RS-2 were studied on the basis of an experiment with ELISA using Hcp effector protein polyclonal antibody. Based on the optical density measurement of Hcp protein (OD_540_) using an indirect ELISA experiment, the drawn standard curve for Hcp protein represented in this study as *y* = 110.4*x* + 0.027 showed a high coefficient of correlation (*R*^2^ = 0.999) for its reliability (Figure 12B). The supernatant of strain RS-2 showed a strong positive ELISA reaction when we used AgNPs at a concentration of 20 μg/ml, however, there was a negative reaction (P/N ≤ 1.5) in the absence of AgNPs for the culture broth of strain RS-2 (Figure 12C). According to a standard curve, colorimetric analysis showed that the concentration of Hcp in the sample treated with AgNPs and positive control was 0.015 and 0.012 mg/ml, respectively. Obviously, these data indicated that AgNPs had an effect on the secretion of Hcp proteins in strain RS-2.

## Discussion

Biologically synthesized AgNPs have been reported to be promising therapeutic molecules with significant antimicrobial and antiviral activities (Kim et al., 2007; Rogers et al., 2008; Galdiero et al., 2011; Oves et al., 2013; Bhuyan et al., 2015; Aziz et al., 2016; Zhang et al., 2016). Although many nanoparticles have been successfully synthesized using microorganisms and plants, searching new nanoparticles with precise biological, physical, and chemical features is still at the cutting edge of nanoscience research. *Phyllanthus* has a noteworthy variety of forms of growth and the fruits are widely used in several preparations of traditional medicine due to its rich antioxidant, anti-aging, antipuretic, and anti-inflammatory properties and the potential sources of naturally occurring phytochemcals in the fruit extract (Ramesh et al., 2015; Manikandan et al., 2017). In this study, synthesis and characterization of AgNPs from *P. emblica* fruit extract is reported, which may benefit from ecological and economical aspects. Additionally, synthesized AgNPs have greatly affected the growth of bacteria, integrity of cell, biofilm production, swarming motility and the secretion of Hcp effectors of Ao strain RS-2. Overall, this study reported green-synthesized AgNPs mediated by *P. emblica* plant extract and demonstrated its antibacterial activity and mechanism against the pathogen of bacterial rice brown stripe.

It has been well described that biosynthesis of nanoparticles employing the use of plant extract is a noble strategy for biosynthesis reaction because of their nontoxic properties and thus provide natural capping agents (Sharma et al., 2009; Rai and Ingle, 2012; Oves et al., 2013, 2018; Prasad, 2014; Aziz et al., 2016, 2019; Qayyum et al., 2017). Results here showed that synthesis of AgNPs occurred after exposing silver nitrate to *P. emblica* fruit extract even at different ratios and hence, the change in dark brown color was observed due to the succession of nanoparticles formation, which has been further justified by ultraviolet–visible spectroscopy. Biosynthesized AgNPs had a strong band of absorption at 430 nm, due to its SRP attributes. Here, the intensity of the color change from light yellowish to dark brown is directly related to the quantity of the extract and incubation period, and this is probably because of the stimulation of longitudinal plasmon vibrations and AgNO_3_ reduction (Kumar and Mamidyala, 2011; Yamal et al., 2013; Manikandan et al., 2017). Therefore, in agreement with earlier reports (Sosa et al., 2003; Tran et al., 2013; Manikandan et al., 2017),increasing the absorption point with an increase extract ratio is a reliable criterion, thus indicating the synthesis of symmetrical nanoparticles. With the intention to identify the key factors for the silver ions (Ag^+^) reduction into AgNPs (Ag^0^) in the aqueous extract of *P. emblica* fruits, FTIR analyses have been carried out. In case of AgNPs, a shift in the absorbance peak with variable band intensity was observed at different points when compared with control fruit extract. *P. emblica* fruit extract based AgNPs spectra revealed different absorption bands ranging from 519 to 3404 cm^−1^. This predicts the presence of possible biomolecules that are involved in reduction and stabilization of silver ions (Ag^+^) to AgNPs (Ag^0^) present in aqueous fruit extract. In agreement with previous reports, the FTIR spectrum analysis in this study detected several absorption peaks, specifically for N–H stretching vibrations, indicating strong hydrogen bonding, and C = O extension vibrations attributed to carboxylic acids, ketones, and aldehydes, which were linked to the silver ions reduction leading to nanoparticles stabilization because of oxidizing the hydroxyl radical (Selvi et al., 2016; Manikandan et al., 2017; Qayyum et al., 2017; Oves et al., 2018; Aziz et al., 2019). Different peaks at two–theta value in XRD results revealed the presence of AgNPs having varied face-centered cubic (fcc) silver planes, while the peaks at 2Θ value of 38 degrees was the sharpest and the (111) plane therefore appears to be preferentially similar to the supporting substratum surface. Similar results were also shown in other studies (Mahmoud et al., 2016; Manikandan et al., 2017; Oves et al., 2018; Aziz et al., 2019). The TEM and HRTEM images of biosynthesized AgNPs showed that most of the particles were nearly spherical in shape and crystalline in nature, particularly, the HRTEM images showed the presence of lattice fringe corresponds to Ag plane (Soman and Ray, 2016). In our study, we have observed a few traces of AgNPs clusters that may contribute to particle size variation. On close observation, we can clearly see that the clusters have been formed when the individual particles have clustered together. Various sizes of spherical AgNPs with crystallization of bio-organic compounds were reported by previous studies by TEM and SEM analysis (Elbeshehy et al., 2015; Mahmoud et al., 2016; Ghiuţă et al., 2018; Oves et al., 2018; Aziz et al., 2019). Moreover, the corresponding SAED pattern of the synthesized AgNPs depicted the crystalline structure of the particles, which was again confirmed by the energy dispersive X-ray analysis (EDX) results obtained. The EDX signals confirmed an existence of the silver element in the synthesized AgNPs with a peak optical absorption range, which has been observed in the biosynthesized AgNPs using *P. acidus* and *Solanum xanthocarpum* fruit extract (Amin et al., 2012; Manikandan et al., 2017). Metallic silver nanocrystals generally show typical EDX signals, which is typical for the absorption of metalic silver (Park et al., 2007). EDX peaks from C, O, and Cl may be caused due to the carbon coated copper grid or by the emission of X- rays from proteins and enzymes of fruit extract (Manikandan et al., 2017), while the nanoparticles can be adhered to either by free amino groups or cysteine residues (Mandal et al., 2005).

Synthesized AgNPs using plants or microorganisms are well-known approach for the development of safe and competent control strategies against resistant bacteria (Kim et al., 2007; Oves et al., 2013; Elbeshehy et al., 2015; Aziz et al., 2016; Zhang et al., 2016; Manikandan et al., 2017). *In vitro* results showed that AgNPs synthesized by the fruit extract of *P. emblica* had effective antibacterial activity against strain RS-2 with the inhibition zone of 27–1.96 cm, while the growth of Ao strain RS-2 was dependent on the concentration of nanoparticles. In addition, significant *in vitro* inhibition of bacterial growth (OD_600_ value) using the dosages of 20 and 30 μg/ml AgNPs were observed and the difference was not obvious, suggesting a MIC of 20 μg/ml AgNPs against strain RS-2. Earlier reports also showed that AgNPs have a concentration-dependent inhibitory effect on a wide range of pathogenic bacteria (Aziz et al., 2016; Mahmoud et al., 2016; Qayyum et al., 2017; Oves et al., 2018). These results are also partly explained due to the smallest AgNPs (having an average diameter of 39 nm), which has been shown a strong inhibitory effect against many Gram-negative bacteria elsewhere (Pal et al., 2007; Singh et al., 2015). Furthermore, similar to previous reports (Lara et al., 2010; Zarei et al., 2014; Qayyum et al., 2017; Oves et al., 2018), this report also showed that bacterial survival in AgNPs solution was affected by the incubation time, while after 6 h exposing, AgNPs concentration of 20 μg/ml had an about 4 log reduction in the viable population of Ao strain RS-2 compared with control.

In addition, TEM micrographs have also shown the different morphological changes that occurred in Ao strain RS-2 upon exposure of AgNPs (20 μg/ml). It has been reported that green nanoparticles have the ability to attach the bacterial cell membrane more quickly and strongly compared to chemically synthesized nanoparticles that support its better antibacterial action (Parashar et al., 2011). Therefore, TEM was used to see how AgNPs interact with bacterial cells. With the addition of the synthesized AgNPs, the reduction of bacterial numbers and further bacterial death could be caused by the damage of bacterial cell integrity and leakage of cytoplasm. The bacteriostatic effect of AgNPs can be justified by the analysis of bacterial live/dead cell staining results. According to the previous studies, AgNPs directly altered cellular processes, including permeability, transport of electron, osmoregulation, and respiration, perhaps because of the attachment of Ag^+^ ions with the negatively charged cell-surface. Consequently, it causes the release of bacterial DNA (Marambio-Jones and Hoek, 2010; Rajeshkumar and Malarkodi, 2014), which was clearly revealed from the TEM figures. Qayyum et al. (2017) has been demonstrated that AgNPs interaction with bacterial cells led to the production of ROS, which might be partially justified the distortion of bacterial membranes or lysis of bacterial cells that ultimately led to death of cells. Therefore, it is reasonable to assume that antimicrobial activity of AgNPs can be attributed, at least in part, to the damage of the membrane and cytoplasm of Ao.

A noticeable finding of this study is that AgNPs inhibited the swarming motility and biofilm formation of Ao strain RS-2 in comparison with control, indicating that inhibitory effect of AgNPs is partly due to the disruption of flagella and interference with the biofilm formation. Moreover, it has been reported that green AgNPs interact with bacterial cells and produced ROS, which causes protein denaturation and other macromolecules damage and improper expression of the bacterial virulence factor, including inhibition of biofilm (Qayyum et al., 2017). Previous studies demonstrated that bacterial movement was directly linked to the growth of bacteria, biofilm formation and pathogenesis (Bahar et al., 2010; Liu et al., 2012). Swarming ability of bacteria has indeed been shown to be a decisive factor in colonizing the host for successful infection through attachment and chemotaxis via type IV pili (Liu et al., 2012; Ogunyemi et al., 2019). Besides, biofilm formation was thought to play a vital role in the virulence of plant pathogenic bacteria by employing several mechanisms such as (i) increasing the potential to survive in hazardous conditions and limited nutrient availability, (ii) emerging resistance to plant-derived antibacterial compounds and/or, (iii) stimulating colonization of the host (Mansfield et al., 2012; Bogino et al., 2013). Moreover, swarming is crucial for the various phages of biofilm development, such as hunting for a favorable locale, surface adherence, structural disassembly, and discharge from the matrix of biofilm (Klausen et al., 2003). Additionally, our previous study found that the production of exopolysaccharide (EPS), a key component of the biofilm complex is strongly associated with virulence factors in Ao strain RS-1 (Zhang et al., 2017). According to the findings here, several studies have also been shown that AgNPs was able to affect the swarming ability and biofilm formation, resulting in less virulence (Vyshnava et al., 2016; Qayyum et al., 2017; Alavi and Karimi, 2018).

Results of the ELISA showed that Ao strain RS-2 treated with AgNPs caused increased secretion of Hcp protein as compared to the control (without AgNPs).These results are pretty congruent with the observations of Dong et al., 2016), who reported that camptothecin, a monoterpenoid indole alkaloid antimicrobial compound, caused the up-regulated expression of *hcp* and the increased secretion of Hcp in live Ao strain RS-2 detected on ELISA analysis. Indeed, Hcp are thought to be the components or effectors proteins as T6SS-hallmark components in many Gram-negative bacteria (Mougous et al., 2006; Wu et al., 2008; Masum et al., 2017). Moreover, recent studies have also reported that T6SS play a key function in several virulence-related attributes such as growth of bacteria, production of biofilm and extracellular polymeric substances, EPS production, survival, and rigidity to a variety of stimuli (Burtnick and Brett, 2013; Cui et al., 2015; Masum et al., 2017; Hu et al., 2018). Additionally, according to the results achieved in this study, Ho et al. (2013) also observed that T6SS activity was strongly induced due to damaged membrane, therefore, it is reasonably to infer that the increased secretion of Hcp effectors proteins in strain RS-2 could be partly attributed to membrane disruption.

The use of biogenic AgNPs in the protection of crop diseases offers an excellent promise in insect and pathogens management as an alternative to chemically produced pesticides. AgNPs are remarkably effective against phytopathogens with low toxicity and lead to broad spectrum of applicability such as in pesticidal, antiviral, antifungal, antibacterial as well as nematicidal activities (Gupta et al., 2018). It opens up a new tool for disease management, rapid disease detection and reducing nutrient losses in fertilization by an optimized nutrient management. Moreover, AgNPs can be used as a foliar spray to halt the growth of fungi, molds, rot and several other plant diseases due to its quite stable and highly dispersive in water solution. However, they may have some limitations of its bactericidal nature able to kill beneficial microbes or it may be no effect on the beneficial microbes. Research on the application of nanoparticles in agriculture is still in the early stages, especially with regard to their interventions with microorganisms that are beneficial for agriculture. Because of the lack of effective environmental safety protocols, only *in vitro* studies have been reported so far and *in vivo* studies are still not adequately documented. Preliminary we tested the impact of AgNPs on plant growth promoting bacteria (PGPBs), including *Bacillus amyloliquefaciens* strain D16 isolated from rice and *Paneabacillus polymyxa* strain SX3 from cotton in our laboratory, and hence, we did not observe a significant effect on PGPBs upon exposure the concentration of 5–20 μg/mL (data not shown). Nonetheless, other studies demonstrated that PGPBs are inhibited upon exposure to AgNPs but the toxic effects above a certain concentration (mostly ≥ 100ppm) (Gupta et al., 2018; Mahawar and Prasanna, 2018). However, extrapolation of our observations to more general cases is limited because of *in vitro* evaluation. On the other hand, it has been reported that low dose of AgNPs can up-regulate the nitrogen fixation genes and increases the functioning of arbuscular mycorrhizal fungi in the plant rhizosphere (Mahawar and Prasanna, 2018). In general, the effect of nanoparticles (NP) interactions with plants or microbes is largely depended on “nano-specific” (type, size, surface charge), doses, species of plant or microbe, and media. Furthermore, there is immense research scope in this unexplored, promising and challenging area and hence, a clear picture of the agro-ecological consequences of AgNPs would necessitate more in-depth perceptive studies in interaction of plant-microbe-nanoparticle systems.

## Conclusion

In conclusion, this study clearly provides an economical, environmental friendly, and straightforward reproducible approach in AgNPs synthesis employing *P. Emblica* fruit extracts as a reducing, stabilizing, and capping agent. The biosynthesized AgNPs were characterized thoroughly by UV-Visible and Energy Dispersive X-Ray Spectroscopy, X-Ray diffraction, FTIR, TEM, HRTEM and SEM. The FTIR results found several phytochemicals responsible for the rapid reduction of ions, leading to AgNPs formation. Especially, hydroxyl groups oxidation of hydrolysate, which likely stimulated the formation of nanoparticles. In the reaction mixture, biosynthesized AgNPs have been detected as mono-dispersed, rather stable, of comparatively smaller in shape and were adhered with an organic layer, in which proteins participated. Furthermore, this study clearly demonstrated that bacterial growths of Ao strain RS-2 were inhibited by the synthesized AgNPs, while effects varied with the period of incubation and applied concentration. Hence, it is noticeably observed that the MIC of AgNPs at 20 μg/ml was able to affect bacterial growth, cell survival, biofilm formation, and swarming ability. ELISA experiment demonstrated that AgNPs resulted in increased secretion of Hcp proteins in Ao strain RS-2, which might be justified by the damaged membrane in reference to TEM images and results of live/dead cell staining assays. The bacteriostatic effect of AgNPs is generally achieved due to direct interaction between AgNPs and bacterial cells, which caused the destruction of biofilm and cell membrane and released intracellular materials from bacteria. In addition, until now, only *in vitro* studies have been reported and *in vivo* studies are not yet adequately documented, because of the lack of effective environmental safety protocols. However, our results could be used in the future to detect and catalog AgNPs with antibacterial properties to protect crops. Altogether, this project clearly showed the antimicrobial potential of biosynthesized AgNPs to control the pathogen of rice bacterial brown stripe.

## Author Contributions

MMM, MMS, and BL made a significant contribution to the design of the experiments. MMM, YZ, and KA participated to perform the experiments. MMM, MMS, KA, and YA contributed appreciably to the collection and analysis of data. YA, EI, CY, and BL provided chemicals, materials, and tools for analysis. MMM, BL, and WQ had substantial contributions to the interpretation of data and preparing the manuscript. All authors reviewed and approved final manuscript.

## Conflict of Interest Statement

The authors declare that the research was conducted in the absence of any commercial or financial relationships that could be construed as a potential conflict of interest.
